# Prognostic value of cerebral tissue oxygen saturation during neonatal extracorporeal membrane oxygenation

**DOI:** 10.1371/journal.pone.0172991

**Published:** 2017-03-09

**Authors:** Marie-Philippine Clair, Jérôme Rambaud, Adrien Flahault, Romain Guedj, Julia Guilbert, Isabelle Guellec, Amélie Durandy, Maryne Demoulin, Sandrine Jean, Delphine Mitanchez, François Chalard, Chiara Sileo, Ricardo Carbajal, Sylvain Renolleau, Pierre-Louis Léger

**Affiliations:** 1 Department of Neonatal and Pediatric intensive care unit, Trousseau Hospital, AP-HP, Paris, France; 2 Laboratory of Central Neuropeptides in the Regulation of Body Fluid Homeostasis and Cardiovascular Functions, Center for Interdisciplinary Research in Biology (CIRB), INSERM, U1050, Paris, France; 3 CNRS, UMR 7241, Paris, France; 4 Department of Emergency medicine, Trousseau Hospital, AP-HP, Paris, France; 5 Department of Neonatology, Trousseau Hospital, AP-HP, Paris, France; 6 Department of Pediatric Radiology, Trousseau Hospital, AP-HP, Paris, France; 7 UPMC Pierre et Marie Curie University, Paris VI, France; 8 Department of Pediatric intensive care unit, Necker Hospital, AP-HP, Paris, France; Universita degli Studi di Roma La Sapienza, ITALY

## Abstract

**Objectives:**

Extracorporeal membrane oxygenation support is indicated in severe and refractory respiratory or circulatory failures. Neurological complications are typically represented by acute ischemic or hemorrhagic lesions, which induce higher morbidity and mortality. The primary goal of this study was to assess the prognostic value of cerebral tissue oxygen saturation (StcO_2_) on mortality in neonates and young infants treated with ECMO. A secondary objective was to evaluate the association between StcO_2_ and the occurrence of cerebral lesions.

**Study design:**

This was a prospective study in infants < 3 months of age admitted to a pediatric intensive care unit and requiring ECMO support.

**Measurements:**

The assessment of cerebral perfusion was made by continuous StcO_2_ monitoring using near-infrared spectroscopy (NIRS) sensors placed on the two temporo-parietal regions. Neurological lesions were identified by MRI or transfontanellar echography.

**Results:**

Thirty-four infants <3 months of age were included in the study over a period of 18 months. The ECMO duration was 10±7 days. The survival rate was 50% (17/34 patients), and the proportion of brain injuries was 20% (7/34 patients). The mean StcO_2_ during ECMO in the non-survivors was reduced in both hemispheres (p = 0.0008 right, p = 0.03 left) compared to the survivors. StcO_2_ was also reduced in deceased or brain-injured patients compared to the survivors without brain injury (p = 0.002).

**Conclusion:**

StcO_2_ appears to be a strong prognostic factor of survival and of the presence of cerebral lesions in young infants during ECMO.

## Introduction

Extracorporeal membrane oxygenation (ECMO) is an artificial means of providing oxygenation and carbon dioxide (CO_2_) elimination in patients who have refractory acute respiratory or hemodynamic failure. It temporarily supports heart and lung function, which may provide time for injured lungs to recover and enable the treatment of underlying diseases. Neurological complications such as both hemorrhagic or ischemic stroke are frequent (10% to 20% according to a series) [1]. Several mechanisms can play a role in the onset of a brain injury. First, pre-ECMO hemodynamic disturbances (sepsis, acidosis, lactate level) can alter cerebral circulation [2]. Second, cerebral blood flow (CBF) can decrease after carotid canulationcanulation [3]. Third, jugular canulation can induce high venous pressures in the superior vena cava, which can impact cerebral venous drainage [4]. Finally, these intracranial pressure changes alter cerebral autoregulation after initiating ECMO and thus induce a decrease in CBF [5].

Currently, certain techniques provide insight into cerebral perfusion during ECMO. Near-infrared spectroscopy (NIRS) measures cerebral tissue oxygenation saturation (StcO_2_) [6]. StcO_2_ depends on oxygen blood transportation as well as CBF [7]. During pediatric cardiac surgery, StcO_2_ desaturation has been found to occur in 70% of patients [8] and has been inversely correlated with morbidity and mortality [9]. During the canulationcanulation procedure, low StcO_2_ has been related to a decrease in cerebral perfusion [10]. Zulueta et al. have shown that intraoperative high cerebral StcO_2_ desaturations during cardiac surgery were correlated with macrocirculatory changes involving a low cardiac index [11]. Their results confirmed the impact of systemic hemodynamic dysfunction on cerebral circulation. NIRS monitoring during cardiac surgery allowed for the detection of cerebral ischemia when a StcO_2_ decrease was associated with acute hypotension [12]. Current evidence indicates that the systemic events during ECMO can induce the onset of cerebral ischemia [13, 14] and neurodevelopmental outcomes [15] that are detected by a decrease in StcO_2_.

Continuous StcO_2_ monitoring can measure cerebral hemodynamic changes if no hypoxic events or rapid hemoglobin changes occur. Nevertheless, no study has assessed the continuous monitoring of StcO_2_ during prolonged ECMO in neonates and young infants.

We hypothesized that low cerebral tissue saturation (StcO_2_) during prolonged pediatric ECMO can be implicated in brain ischemic lesions and thus be associated with the rate of survival. The main goals of this study were to evaluate the prognostic role of StcO_2_ on survival and its association with the occurrence of brain injuries.

## Patients and methods

### Study design

This observational study was conducted from September 2012 to March 2014 at the Pediatric Intensive Care Unit (PICU) of the Trousseau University Hospital in Paris. This PICU is also a neonatal and pediatric ECMO center and has 10 pediatric and 8 neonatal beds. This study included all neonates and young infants under 3 months of age assisted by ECMO for refractory respiratory and/or circulatory failures according to the following criteria [16]: refractory hypoxemia with an oxygenation index (OI) > 40 during more than 6 hours or an OI > 30 for more than 12 hours; an alveolar-arterial oxygen difference (AaDO_2_) > 620 mm Hg; a PaO_2_ < 40 mmHg for more than 6 hours; refractory hypercapnia with respiratory acidosis with a pH < 7.1; refractory septic shock; and refractory cardiogenic shock. No cardiac surgery patients were included in this study. In veno-venous (V-V) ECMO, canulation was surgically performed at the right internal jugular vein and/or femoral vein. In veno-arterial (V-A) ECMO, venous canulation was performed at the right internal jugular vein and arterial canulation at the right common carotid artery. Pediatric circuits with a centrifugal pump (Rotaflow console, Maquet, Hirrlingen, Germany) or non-occlusive roller pump (A100 console, Rhône-Poulenc, Paris, France) were used. Membrane oxygenators used were either Quadrox-i pediatric (Maquet, Germany) or Hilite 800 LT (Medos, Stolberg, Germany).

The continuous cerebral tissue oxygen saturation was recorded with an Invos® device (Covidien medical, Medtronics, Minneapolis, USA). Two sensors were placed on both temporo-parietal regions. StcO_2_ recording began within the first 6 hours after initiation of ECMO and ended at the discontinuation of ECMO support.

Data were gathered at the end of each recording. The INVOS Analytics Tool software was used to analyze all the data; the following main parameters were measured: mean StcO_2_, the duration of oxygen desaturation below the threshold of 20% from baseline and the duration of oxygen desaturation below the StcO_2_ value of 50%. The mean StcO_2_ value typically fluctuated between 60%-70% in normal conditions.

The calculation of the oxygenation index [(MAP x FiO_2_)/PaO_2_] was performed by measuring the PaO_2_ of arterial blood gas before placement on ECMO. If an arterial blood gas was not available, we used a specific scale for estimating the PaO_2_ with the pulse arterial oxygen saturation according to the method described by Severinghaus [17].

Magnetic resonance imaging (MRI) was performed after the discontinuation of ECMO and withdrawal of cannulas in all surviving patients. Brain injuries were defined as ischemic or hemorrhagic lesions based on the extent and topography of the affected regions.

The French Research Ethics Committee (Comité de Protection des Personnes Ile-de-France) approved this study without any further requirements owing to the lack of interventional procedures. Parents were informed of the protocol in progress and were able to present their objection to the collection of any data regarding their infants. Consents to participate are not applicable because it is an non-invasive observational study.

### Statistical analysis

The continuous variables were analyzed using a non-parametric Wilcoxon test, whereas categorical data were analyzed by Fisher's exact test. Statistical significance was defined as p<0.05. Statistical analyses were performed with R programming software.

## Study objectives

The primary objective of the study was to assess the association between StcO_2_ and survival during pediatric prolonged ECMO.

There were two secondary objectives: first, to compare the StcO_2_ in brain-injured or deceased patients versus survivors without injury; and second, to compare V-V ECMO patients with VA-ECMO patients.

## Results

### Description of the population

Thirty-four patients were enrolled in our study (Table 1). The mean ± SD age was 24 ± 36 days, the mean birth weight was 3480 ± 815 g and the mean gestational age was 39 ± 2 weeks. The etiologies were diverse: diaphragmatic hernia (12 patients), meconium aspiration syndrome (7), severe bronchiolitis (4), refractory septic shock (4), opportunistic infections (4), and malignant pertussis (3). Before ECMO, the disease severity parameters were OI = 39 ± 16, AaDO_2_ = 520 ± 178 mmHg, PaO_2_/FiO_2_ = 79 ± 50, pH = 7.2 ± 0.16, and PaCO_2_ = 50 ± 16 mmHg. Thirty-two patients (94%) had been treated with vasopressors, and 10 patients (29%) had been treated with inotropic drugs. Seventeen of the patients were on V-V ECMO, and 17 infants were on V-A ECMO. The mean duration of ECMO was 10±7 days.

**Table 1:** Patient characteristics.

	Patients [n = 34]
**Age *(days)***	24 ± 36
**Birth weight *(g)***	3480 ±815
**Gestation age *(weeks)***	39 ± 2
**Male/ Female *(n)***	17/17
**Survival (*n*, *%)***	17 (50)
**Cerebral injury *(n*, *%)***	7 (18)
**ECMO indications *(n*, *%)***	
Congenital diaphragmatic hernia	12 (36)
Meconium aspiration syndrome	7 (21)
Bronchiolitis	4 (12)
Septic shock	4 (12)
Opportunist infection	4 (12)
Pertussis	3 (9)
**Pre-ECMO parameters**	
Ventilation *(days)*	17 ± 11
Tidal volume *(mL/kg)*	7 ± 2,4
OI	39 ± 16
PEEP *(cmH2O)*	6 ± 1,8
FiO_2_ *(%)*	91 ± 18
HFO *(n*, *%)*	15 (56)
pH	7,2 ± 0,16
PaCO_2_ *(mmHg)*	50 ± 16
Lactate *(mmol/L)*	3,6 ± 2,5
AaDO_2_ *(mmHg)*	520 ± 178
PaO_2_/FiO_2_	79 ±50
**Pre-ECMO treatment**	
Nitric oxide *(n*, *%)*	24 (71)
Vasopressor *(n*, *%)*	32 (94)
Inotropic *(n*, *%)*	10 (29)
Fluid expansions *(n bolus)*	1,8 ± 1,5
**V-A/V-V ECMO *(n)***	17/17
**ECMO duration *(days)***	10 ± 7

Data are given as mean ± standard deviation, or number of patients (*n*) followed by the percent (%); PEEP = positive end-expiratory pressure; OI = oxygenation index, HFO = high frequency ventilation; V-A = veno-arterial, V-V = veno-venous, ECMO = extracorporeal membrane oxygenation; AaDO_2_ = Alveolo-arterial O_2_ difference, PaCO_2_ = CO_2_ arterial pressure_;_ PaO_2_ = O_2_ arterial pressure.

No differences in demographic characteristics were observed between survivors and non-survivors (Table 2). However, other significant differences were observed. The pre-ECMO mean OI was higher in survivors than in non-survivors (51 ± 15 vs 30 ± 18, p = 0.02), and the pre-ECMO PaCO_2_ was higher in survivors than in non-survivors (57 ± 18 mmHg vs 43 ± 9 mmHg, p = 0.02). There were more patients who received V-A ECMO among non-survivors than survivors (71% vs 29%, p = 0.04). Half of the patients were discharged alive from the intensive care unit.

**Table 2:** Characteristic between survivors versus non survivors.

	Survivors [n = 17]	Non Survivors [n = 17]	*p*
**Age *(days)***	20 ± 33	28 ± 39	0,22
**Birth weight *(g)***	3700 ± 900	3260 ± 640	0,3
**Gestational age *(weeks)***	40 ± 1,5	39 ± 1,9	0,09
**Ventilation parameters**			
Pre ECMO ventilation *(days)*	20 ± 13	14 ± 10	0,22
Tidal volume *(mL/kg)*	7,3 ± 1,9	6,6 ± 2,9	0,15
OI	51 ± 15	30 ± 18	0,02
PEEP *(cmH2O)*	7 ± 1	6 ± 2	0,22
FiO_2_ *(%)*	90 ± 20	92 ± 17	0,67
HFO *(n*, *%)*	7 (41)	8 (47)	1
**Pre-ECMO parameters**			
pH	7,2 ± 0,17	7,2 ± 0,14	0,41
PaCO_2_ *(mmHg)*	57 ± 18	43 ± 9	0,02
Lactate *(mmol/L)*	3,6 ± 3,2	3,4 ± 2	0,82
AaDO_2_ *(mmHg)*	567 ± 95	454 ± 230	0,28
PaO_2_/FiO_2_	76 ± 38	84 ± 65	0,81
**Pre-ECMO treatments**			
Nitric oxide *(n*, *%)*	12 (71)	12 (71)	1
Vasopressor *(n*, *%)*	16 (95)	16 (95)	1
Inotropic *(n*, *%)*	5 (29)	5 (29)	1
Fluid expansions *(n bolus)*	1,6 ± 1,6	2 ±1,6	0,41
**V-A ECMO *(n*, *%)***	5 (29)	12 (71)	0,04
**Total ECMO duration *(days)***	10 ± 7	9 ±7	0,64

Data are given as mean ± standard deviation, or number of patients followed by the percent. PEEP = positive end-expiratory pressure; OI = oxygenation index, HFO = high frequency ventilation; V-A = veno-arterial, ECMO = extracorporeal membrane oxygenation; AaDO_2_ = Alveolo-arterial O_2_ difference, PaCO_2_ = CO_2_ arterial pressure_;_ PaO_2_ = O_2_ arterial pressure.

Seven patients of the 17 survivors (41%) presented brain lesions on cerebral MRIs. All injuries were due to ischemic stroke. Ischemic lesions were located in the left hemisphere (4 patients), in the right hemisphere (1) and in both hemispheres (2).

The mean temporo-parietal StcO_2_ in survivors and non-survivors were 69 ± 8% and 54 ± 14% (p = 0.0008), respectively, in the right hemisphere and 67 ± 7% and 56 ± 16% (p = 0.03), respectively, in the left hemisphere (Table 3). The mean StcO_2_ was significantly reduced in the right hemisphere (56 ± 14%) among brain-injured and deceased patients compared to survivors without brain injury (70 ± 8%, p = 0.002). Nevertheless, no significant difference was observed in the left hemisphere (59 ± 7% vs 66±6%, p = 0.14) (Table 4).

**Table 3:** Cerebral tissue oxygen saturation between survivors and non survivors.

	Survivors [n = 17]	Non survivors [n = 17]	*p*
**Right temporo-parietal**			
StcO_2_ *(mean %)*	69 ± 8	54 ± 14	0,0008
StcO_2_ <50% *(min)*	3 ± 7	31 ± 27	0,0001
ΔStcO_2_ >20% *(min)*	3 ± 7	28 ± 25	0,0002
**Left temporo-parietal**			
StcO_2_ *(mean %)*	67 ± 7	56 ± 16	0,03
StcO_2_ <50% *(min)*	5 ± 7	22 ± 28	0,01
ΔStcO_2_ >20% *(min)*	6 ± 7	20 ±27	0,02

StcO_2_: mean tissue oxygen saturation on the total recording time during ECMO duration. StcO_2_ <50%: mean time spent below 50%. Δ StcO_2_ > 20%: mean time spent below 20% of the mean StcO_2_. Data are given as mean ± standard deviation.

**Table 4:** Cerebral tissue oxygen saturation between brain injured and non-survivors patients versus patients without brain injury.

	Survivors without brain injury [n = 13]	Non-survivors or brain injury [n = 21]	*p*
**Right temporo-parietal**			
StcO_2_ *(mean %)*	70 ± 8	56 ± 14	0,002
StcO_2_ <50% *(min)*	3 ± 8	26 ± 27	0,001
ΔStcO_2_ >20% *(min)*	4 ± 8	23 ± 25	0,003
**Left temporo-parietal**			
StcO_2_ *(mean %)*	66 ± 6	59 ± 17	0,14
StcO_2_ <50% *(min)*	5 ± 9	19 ± 27	0,05
ΔStcO_2_ >20% *(min)*	6 ± 8	17 ± 8	0,04

StcO_2_: mean cerebral tissue oxygen saturation recording time during ECMO duration. StcO_2_ <50%: mean time spent below 50%. Δ StcO_2_ > 20%: mean time spent below 20% of the mean StcO_2_. Data are given as mean ± standard deviation.

The duration of oxygen desaturation (time spent below 50% StcO_2_ and time spent below 20% of the mean StcO_2_) was significantly longer in non-survivors than survivors (Table 3). The duration of oxygen desaturation was also significantly increased in non-survivors and brain-injured patients compared to survivors without brain injury (Table 4). This significant difference was observed in both hemispheres. The mean StcO_2_ and duration of oxygen desaturation were not different in patients with V-V ECMO compared to V-A ECMO (Table 5).

**Table 5:** Cerebral tissue oxygen saturation between V-V ECMO and V-A ECMO.

	V-V ECMO [n = 17]	V-A ECMO [n = 17]	*p*
**Right temporo-parietal**			
StcO_2_ *(mean %)*	65 ±11	59 ± 16	0,42
StcO_2_ <50% *(min)*	10 ± 17	23 ± 29	0,28
ΔStcO_2_ >20% *(min)*	9 ± 13	21 ± 27	0,28
**Left temporo-parietal**			
StcO_2_ *(mean %)*	62 ± 13	61 ± 15	0,87
StcO_2_ <50% *(min)*	9 ± 17	17 ± 26	0,28
ΔStcO_2_ >20% *(min)*	9 ± 15	15 ± 25	0,33

StcO_2_: mean tissue oxygen saturation on the total recording time during ECMO duration. StcO_2_ <50%: mean time spent below 50%. Δ StcO_2_ > 20%: mean time spent below 20% of the mean StcO_2_. Data are given as mean ± standard deviation. V-A = veno-arterial, V-V = veno-venous.

## Discussion

This is the first study exploring continuous cerebral tissue oxygen saturation during pediatric prolonged ECMO and its association with morbidity and mortality. First, we found that the mean StcO_2_ decreased in non-surviving newborns and young infants during prolonged ECMO. Second, the mean StcO_2_ and the duration of desaturation remained normal in patients without brain injury.

Although the high ECMO mortality was due to the severity of the underlying diseases (ARDS, septic shock), the morbidity was primarily due ischemic or hemorrhagic stroke [18] [19]. In our ECMO experience, the ischemic brain lesions occurred in 20% of newborns [20]. Rollins et al. reported up to 62% of cases that had abnormal MRIs after neonatal ECMO, whereas ultrasound imaging was abnormal in only 24% of the cases [21]. Therefore, although MRI improves the screening of cerebral injuries, earlier detection should lead to improved care.

Neurological injuries during ECMO were closely linked to cerebral perfusion. The decrease of StcO_2_ below 50% during extracorporeal support was correlated with an increased rate of cerebral injuries [22]. In other studies, StcO_2_ monitoring during surgery detected alterations in cerebral hemodynamics [13, 23].

In this study, the duration of oxygen desaturation was longer in brain-injured and deceased pediatric patients. It should be noted, however, that the mean duration of desaturation was only 20 minutes during the entire ECMO period. This duration of desaturation was very short compared to the total ECMO support (a mean of 10 days). It appears unlikely that these desaturating episodes significantly affected the mean StcO_2_. Therefore, the decrease of the mean StcO_2_ could be more related to less intense but more prolonged episodes of cerebral hypoxia and/or ischemia during ECMO.

Our results suggest that continuous StcO_2_ monitoring may be an important approach to predict both mortality and cerebral ischemia. The ischemic stroke events during ECMO were located in the left hemisphere in 70% of the cases [24]. The reasons are likely multifactorial. First, cannula insertions into the internal carotid artery and/or in the right jugular vein may directly impact cerebral perfusion. Jugular canulation rapidly changes the pressures within veins due to decreases in venous drainage. Second, the definitive ligation of the jugular vein prolongs high pressure levels in the right cerebral circulation. Similarly, carotid canulation abolishes right anterograde circulation. Thus, the collateral blood flow between the two hemispheres is profoundly modified after canulations and so may be the major prognostic factor of cerebral ischemia during ECMO. Within this context, the cerebral tissue oxygenation desaturation may become an interesting clinical marker of hypoperfusion.

Our study has some limitations. First, our cohort size was small. Second, only newborns and young infants were included, which limits extrapolations to older children. Third, this was an observational non-interventional study without a control group. Fourth, we did not monitor continuous arterial saturation during the study period. Thus, we cannot exclude that cerebral tissue desaturation was partly due to arterial desaturation and not only to a decrease in CBF. Concerning pre-ECMO parameters, PaCO_2_ was higher before ECMO in surviving patients. PaCO_2_ also has a strong vasodilator effect on cerebral arteries [25]. Hypercapnia may exert an effect on cerebral perfusion during ECMO, increasing cerebral blood flow and subsequently StcO_2_. Arituk *et al*. have shown in 126 patients that cerebral StcO_2_ decreased to 10% when the mean PaCO_2_ decreased from 38 to 30 mmHg without a corresponding alteration of arterial oxygen, lactate, mean arterial pressure or blood flow on the extracorporeal support [26]. The authors concluded that there was a vasoconstriction effect on cerebral arteries. Conversely, StcO_2_ increased from 53% to 63% when PaCO_2_ increased from 31 to 41 mmHg during cardiopulmonary bypass [27]. Thus, it is likely that, in the survivor group, an improvement in cerebral perfusion due to hypercapnia-induced arterial vasodilation may have reduced the ischemic process [28] during ECMO.

## Conclusion

This is the first observational study assessing the prognostic value of cerebral tissue oxygen saturation on survival during pediatric prolonged ECMO. The study also evaluated the relationship between desaturations measured by continuous StcO_2_ monitoring and brain injuries. The mean StcO_2_ was lower in non-survivors and the duration of cerebral tissue oxygen desaturation was higher in non-survivors or in brain-injured newborns and young infants. Future studies should explore the hemodynamic effect of PaCO_2_ on the cerebral collateral arteries during ECMO. Additionally, some neuroprotective strategies should be developed and assessed using target StcO_2_ values.
